# Should I post? The relationships among social media use, emotion recognition, and mental health

**DOI:** 10.3389/fpsyg.2023.1161300

**Published:** 2023-05-23

**Authors:** Emily Scarpulla, Morgan D. Stosic, Adele E. Weaver, Mollie A. Ruben

**Affiliations:** ^1^Department of Psychology, University of Maine, Orono, ME, United States; ^2^Department of Psychology, University of Rhode Island, Kingston, RI, United States

**Keywords:** active social media use, passive social media use, emotion recognition, impression formation, mental health, well-being, social media

## Abstract

**Introduction:**

While increased time spent on social media can be negatively related to one’s overall mental health, social media research often fails to account for what behaviors users are actually engaging in while they are online. The present research helps to address this gap by measuring participants’ active and passive social media behavioral styles and investigates whether and how these two social media behavioral styles are related to depression, anxiety, and stress, and the mediating role of emotion recognition ability in this relationship.

**Methods:**

A pre-study (*N* = 128) tested whether various social media behaviors reliably grouped into active and passive behavioral styles, and a main study (*N* = 139) tested the relationships between social media use style, emotion recognition, and mental health.

**Results:**

While we did not find evidence of a mediating relationship between these variables, results supported that more active social media use was related to more severe anxiety and stress as well as poorer emotion recognition skill, while passive social media use was unrelated to these outcomes.

**Discussion:**

These findings highlight that, beyond objective time spent on social media, future research must consider how users are spending their time online.

## Introduction

Within the past decade, social media usage has skyrocketed, with 302 million users in the United States (U.S.) totaling 90% of the U.S. population as of 2022 (Dixon, 2023). Social media has been defined by Swar and Hameed (2017) as “the websites and online tools that facilitate interactions between users by providing them opportunities to share information, opinions, and interest” (p. 141). These sites include examples such as Facebook, Instagram, Twitter, Snapchat, and TikTok, which serve as some of the most widely used social media platforms today (Dixon, 2023). Working professionals utilize these sites to network for job opportunities, information (both true and false) can now be disseminated at incredible speeds, new friendships and romantic relationships are forged without any physical contact, and for some deemed ‘influencers’, being active on social media serves as a career. While it is clear that the utilization of social media has had a major impact on the world we live in, it is much less clear how social media use impacts individuals’ own well-being. We address this question by examining whether the behaviors people engage in on various social media platforms are related to their depression, anxiety, and stress, and we explore emotion recognition skill, a central social ability, as a potential mediator of this relationship.

### Social media use and mental health

Social media use has been associated with a variety of interpersonal problems such as the inability to develop closeness with others, loneliness, and shyness (Yao and Zhong, 2014; Bian and Leung, 2015), and subsequent mental health outcomes including higher levels of depression, anxiety, and overall psychological distress (Dhir et al., 2018; Reer et al., 2019; Keles et al., 2020). Wright et al. (2013) showed that the number of hours students spent using Facebook during their college studies was related to depression, and Kross et al. (2013) similarly found that the more individuals used Facebook over a 2-week period, the more their life satisfaction declined. These findings are further supported by various meta-analyses that have reported small but significant effect sizes regarding the relationship between social media use and depression between *r* = 0.11 (Yoon et al., 2019; Ivie et al., 2020; Cunningham et al., 2021) and *r* = 0.17 (Vahedi and Zannella, 2021).

#### Active versus passive social media use

Although these various studies suggest that time spent on social media may be positively related to mental health symptoms such as depression or anxiety, simply measuring and discussing time spent on social media loses the nuances of how users are interacting with social media content and, as such, may be responsible for a large deal of heterogeneity found in effect sizes within previous meta-analyses. To address this gap, some studies have begun to explore two types of social media use: active and passive (e.g., Deters and Mehl, 2013; Krasnova et al., 2013; Verduyn et al., 2015; Gerson et al., 2017; Ruben et al., 2021). Burke et al. (2010) were the first to suggest that certain social media activities can be dichotomized in this way, and researchers have since refined this observation by defining active social media use (ASMU) as “targeted one-on-one exchanges” (e.g., private messaging) or “broadcasting (e.g., posting statuses) (Verduyn et al., 2020, p. 33) and passive social media use (PSMU) as “monitoring the online life of other users without engaging in direct exchanges with them” (e.g., scrolling through their news feed) (Verduyn et al., 2020, p. 33).

One prominent hypothesis throughout the literature has been that more ASMU leads to better mental health symptomologies as it elicits support, positive feedback (e.g., likes), and relationship building while PSMU leads to declines in mental health because it induces upward social comparison and envy (Verduyn et al., 2017; Guyer and Vaughan-Johnston, 2020; Verduyn et al., 2020). Yet, other scholars have argued the opposite - that ASMU may be related to poorer mental health (e.g., Kross et al., 2021) due to the replacement of in-person relationships with online relationships (e.g., Kraut et al., 1998; Nie, 2001), posting unrealistic content of oneself (e.g., using filters, editing software, etc.), or even because those who have poorer mental health may simply prefer to engage socially online opposed to in-person. Similarly, some researchers have posited that some types of passive social media behaviors may be related to better mental health outcomes (e.g., Noon and Meier, 2019; Meier et al., 2020; Valkenburg et al., 2022a) due to the positive experience of assimilative (i.e., focus on similarities) social comparison (Crusius et al., 2022), admiration of or inspiration from others (Meier and Schäfer, 2018; Noon and Meier, 2019; Meier et al., 2020), or because people with better mental health simply prefer to use social media in a passive manner. A recent scoping review attempted to explore these two competing hypotheses by examining associations between ASMU and PSMU and well-being/ill-being (Valkenburg et al., 2022b), which may be considered sibling constructs to mental health (Lawson and Robins, 2021). Of the 172 effects that were analyzed, 0% supported the hypothesis that ASMU was related to less ill-being, and less than half (44%) supported the hypothesis that PSMU was related to more ill-being.^1^ Additionally, other meta-analyses examining these associations (Hancock et al., 2019; Liu et al., 2019; Yin et al., 2019) have yielded inconsistent findings.

While these heterogeneous effects could be due to various factors, some have suggested it may be due to researchers’ *measurement* of ASMU and PSMU (Meier and Krause, 2022). Trifiro and Gerson (2019) recently published a commentary regarding the lack of universal validated measures for active and passive use. Their primary critique was that, while there are many different operationalizations of ASMU and PSMU, there is currently only one validated measure of these social media styles which was designed to identify what behaviors Facebook users engage in while they are online (Gerson et al., 2017). Because of the unique features of Facebook, this scale cannot be used to measure social media use styles across a wide variety of social media platforms that have varying functions. Another critique of ASMU/PSMU measurement has been that for other operationalizations of these constructs, items that are conceptually ASMU have been used to measure PSMU (based on factor analysis results). Thus, the primary purpose of the present research is to help further assess the unique relationship between ASMU, PSMU, and mental health by modifying Gerson et al.’s (2017) validated measure of social media use on Facebook to extend across five common social media platforms and examining its relation to three components of mental health: depression, anxiety, and stress.

#### Emotion recognition as a possible mediator

In addition to continuously testing and establishing the relationship between social media use and mental health, it is also important to explore mediators of this relationship. While social media research has explored several important mediators in this relationship such as inspiration (Meier and Schäfer, 2018) and upward comparison (Meier et al., 2020) one such potential mediator we believe is deserving of more attention is individuals’ interpersonal skills, such as their ability to recognize others’ emotional expressions. Emotion recognition ability is central to individuals’ interpersonal communication and functioning (Williams and Gray, 2013), and individuals who lack this skill face difficulties in forming and maintaining social relationships with others, online and in person.

In terms of mental health, previous research has demonstrated that poorer emotion recognition ability is associated with worse mental health symptomologies such as depression (Demenescu et al., 2010; Pereira-Lima and Loureiro, 2015), anxiety (Easter et al., 2005; Demenescu et al., 2010; Pereira-Lima and Loureiro, 2015), and stress (Hänggi, 2004). A meta-analysis by Hall et al. (2009) showed that interpersonal sensitivity, defined as the accurate judgment or recall of others, was negatively related to depression with a small but significant effect of *r* = −0.09. These correlational results therefore suggest that individuals with poorer emotional recognition ability also seem to have poorer mental health.

Substantially less research has explored the link between social media use styles and emotion recognition. According to the *cues-filtered-out* theory (CFO; Culnan and Markus, 1987; Keiser, 1987), an early theory regarding the impact of computer-mediated communication on relationship development, online interactions can lack various nonverbal cues such as a physical appearance, tone of voice, facial expression, gaze, posture, touch, space, and gestures (Kiesler et al., 1984; Siegel et al., 1986). Given that practice and feedback is one of the most crucial processes for interpersonal skill improvement (Blanch-Hartigan et al., 2012; Ruben et al., 2015; Schlegel et al., 2017), this theory would imply that greater time spent on social media might hinder emotion recognition ability as individuals are not given sufficient emotional nonverbal cues to practice decoding. Newer theories such as *social information processing* theory (Walther and Burgoon, 1992) have articulated that given sufficient time online, interactants can develop new interaction patterns to compensate for the loss of nonverbal cues, and thus social media use might be unrelated to emotion recognition ability, or perhaps even be related to greater emotion recognition ability. However, it is also possible that what truly matters is *how* individuals spend their time online. For instance, it is possible that individuals who use social media actively by broadcasting (i.e., encoding) information online may not spend as much time practicing and receiving feedback on their decoding skills (e.g., decoding emotions) and thus would perform worse on tests of emotion recognition. Alternatively, individuals who use social media passively may receive substantial practice decoding the emotional displays of those whose online lives they are monitoring and thus perform better on tests of emotion recognition.

Only one study (Ruben et al., 2021), that we are aware of, has tested this question empirically by measuring participants’ self-identification as an active social media user or a passive social media user.^2^ In this study, participants were asked to rate the following two items on an 11-point Likert scale: “I tend to be an active user, posting frequently” and “I tend to be a passive user, scrolling through posts and photos.” Using these two self-report items, Ruben et al. (2021) demonstrated how ASMU was significantly related to *poorer* performance on the Workplace Interpersonal Perception Skill test (WIPS; Dael et al., 2022), which assesses individuals’ interpersonal perception skills by asking them to watch brief video segments of role-played workplace interactions and answer questions about the interpersonal and emotional characteristics of the scenes. On the other hand, PSMU was significantly related to *greater* performance on the WIPS, and greater performance on an explicit test of emotion recognition (Diagnostic Analysis of Nonverbal Accuracy- 2 Adult Faces; DANVA-2AF; Nowicki and Duke, 1994), where participants were shown static photos of actors posing various emotional expressions. Based on these preliminary results and theorizing, ASMU may be negatively associated with emotion recognition skills and thus related to poorer mental health outcomes, while PSMU may be positively related to emotion recognition skills and thus better mental health outcomes.

### Current study

The primary objective of the current research is to examine, for the first time, the relationships between social media use, mental health, and emotion recognition skill, together. We focus our investigation on three specific facets of mental health that have previously established relationships with time spent on social media (Dhir et al., 2018; Reer et al., 2019; Keles et al., 2020): depression, anxiety, and stress. Additionally, while there are many different possible interpersonal skills to examine in this context, we chose to focus on emotion recognition ability as it is considered a core ability that actively contributes to individuals’ ability to interact and communicate in social situations (Williams and Gray, 2013).

We highlight the notion that *how* one uses social media matters and extend upon previous work by measuring individuals’ ASMU and PSMU. In light of recent critiques regarding the lack of universal measures for active and passive use (Trifiro and Gerson, 2019), we modified an existing measures of active and passive social media behaviors on Facebook (Gerson et al., 2017) to capture behaviors users could engage in across social media platforms, regardless of if they are text-based (e.g., Twitter), image-based (e.g., Snapchat), video-based (e.g., TikTok), or a combination (e.g., Instagram and Facebook). Also in line with Trifiro and Gerson’s recommendations, we took care to ensure each behavioral item was reflecting empirically the social media use style we expected it to conceptually.

Thus, we start by testing the reliability of 12 different generalized social media behaviors to determine which discrete behaviors capture ASMU, and which capture PSMU. We then test these active and passive social media items in a new sample of participants in order to determine whether ASMU and PSMU have differing relationships to emotion recognition skill and mental health. Specifically, we hypothesize two mediation models: (1) a positive relationship between ASMU and mental health (i.e., depression, anxiety, and stress) will be explained by emotion recognition skill and (2) a negative relationship between PSMU and mental health (i.e., depression, anxiety, and stress) will be explained by emotion recognition skill.

## Pre-study

A pre-study was used to test the variance shared among 12 different self-reported social media behavior items adapted from Gerson et al.’s (2017) Passive Active Use Measure (PAUM). The purpose of this pre-study was *not* to create a new measure of social media use. Instead, we hoped to establish whether our modified items pulled from the PAUM mapped onto the active and passive factors they were originally intended to capture. We hypothesized that items assessing the extent that users behave as a third-party observer of what others are doing on social media (i.e., passive behaviors) would share more variance with one another than items assessing the extent that users create their own content for others to see on social media (i.e., active behaviors).

### Method

#### Participants

A sample of *N* = 160 participants were obtained through Amazon’s Mechanical Turk (MTurk) online platform and were compensated $0.25 for a study that took approximately 4 min to complete (*M* = 3.93 min, *SD* = 1.59). Due to participants failing attention check questions, completing the entire survey in an unreasonable amount of time (<1 min) or responding incoherently to a free response question, this sample was reduced to a final sample of *N* = 128. Of these, 79 (61.7%) were male, and 49 (38.3%) were female. Participants identified as White (50.8%), Asian (42.2%), Black (4.7%), American Indian or Alaska Native (0.8%), Native Hawaiian or Pacific Islander (0.8%), or another group not listed (1.6%). The majority of participants did not identify as Hispanic or Latine (75.8%) and the mean age of participants was *M*_age_ = 33.83 (*SD* = 11.76).

#### Procedure

Upon reading and signing the informed consent, participants proceeded to the survey where they indicated which of the following social media applications they currently used: Instagram, Facebook, Twitter, Tik Tok, or Snapchat. They were then redirected to the next part of the survey where they were asked to self-report how often they engaged in various active and passive social media behaviors on each social media platform. Participants then completed several demographic questions.

#### Materials

##### ASMU and PSMU questions

Participants were asked to rate on a 1 (*Not often at all*) to 7 (*Very often*) Likert scale how often they engaged in 12 different social media behaviors on five different social media platforms. Thus, participants completed 60 ratings (twelve behaviors for all five social media platforms) measuring the frequency of their social media behaviors. In the case that a participant did not use a specific social media application, they were able to select, “Do not use this app/app does not have this function.”

Six behavior items were designed to ask how often participants engaged in more *passive* social media behaviors which defined users behaving as a third-party observer of what others are doing on social media such as “Looking at friends’ and strangers’ posts/photos” and “Reading through the comments on other peoples’ posts.” The other six behavior items were designed to ask participants how often they engaged in more *active* social media behaviors which defined behaviors that involved a user creating their own content for others to see on social media such as “Posting stories” and “Commenting on friends’/strangers’ posts.”

### Results

Table 1 shows the means and standard deviations of each of the 12 active and passive social media behavior items, collapsed across the five social media platforms we sampled. For the active social media items, the most common self-reported behaviors were “Direct/personally messaging my friends or strangers” and “Commenting on friends’/strangers’ posts.” For passive behaviors, the most commonly reported behaviors were “Scrolling through my feed” and “Looking at friends’ or strangers’ posts/photos.”

**Table 1:** Descriptive statistics for passive and active social media items for the Pre-Study (*N* = 128) and the Main Study (*N* = 139).

	*M* (SD)	
	Pre-study	Main study
ASMU and PSMU scale items		
Active items		
Direct/personally messaging my friends or strangers	4.23 (1.76)	3.27 (1.60)
Commenting on friends’/strangers’ posts	4.05 (1.89)	2.90 (1.41)
Sending posts on the app to other users	3.92 (1.87)	2.87 (1.53)
Editing photos I intend to post/drafting words I intend to post	3.89 (1.89)	2.71 (1.65)
Creating content for others to look at	3.78 (1.82)	2.44 (1.52)
Posting stories	3.69 (1.96)	2.23 (1.45)
Passive items		
Scrolling through my feed	4.99 (1.56)	4.76 (1.46)
Looking at friends’ or strangers’ posts/photos	4.97 (1.40)	4.79 (1.37)
Reading through the comments on other people’s posts	4.57 (1.65)	4.06 (1.51)
Looking through the explore page^a^	4.48 (1.74)	–
Watching stories others have posted	4.46 (1.77)	3.66 (1.76)
Looking through posts related to a particular hashtag^a^	3.79 (1.89)	–

1 = Not often at all and 7 = Very often. ^a^Items excluded from the main study due to concerns with reliability.

#### Factor analysis

**Figure 1:**
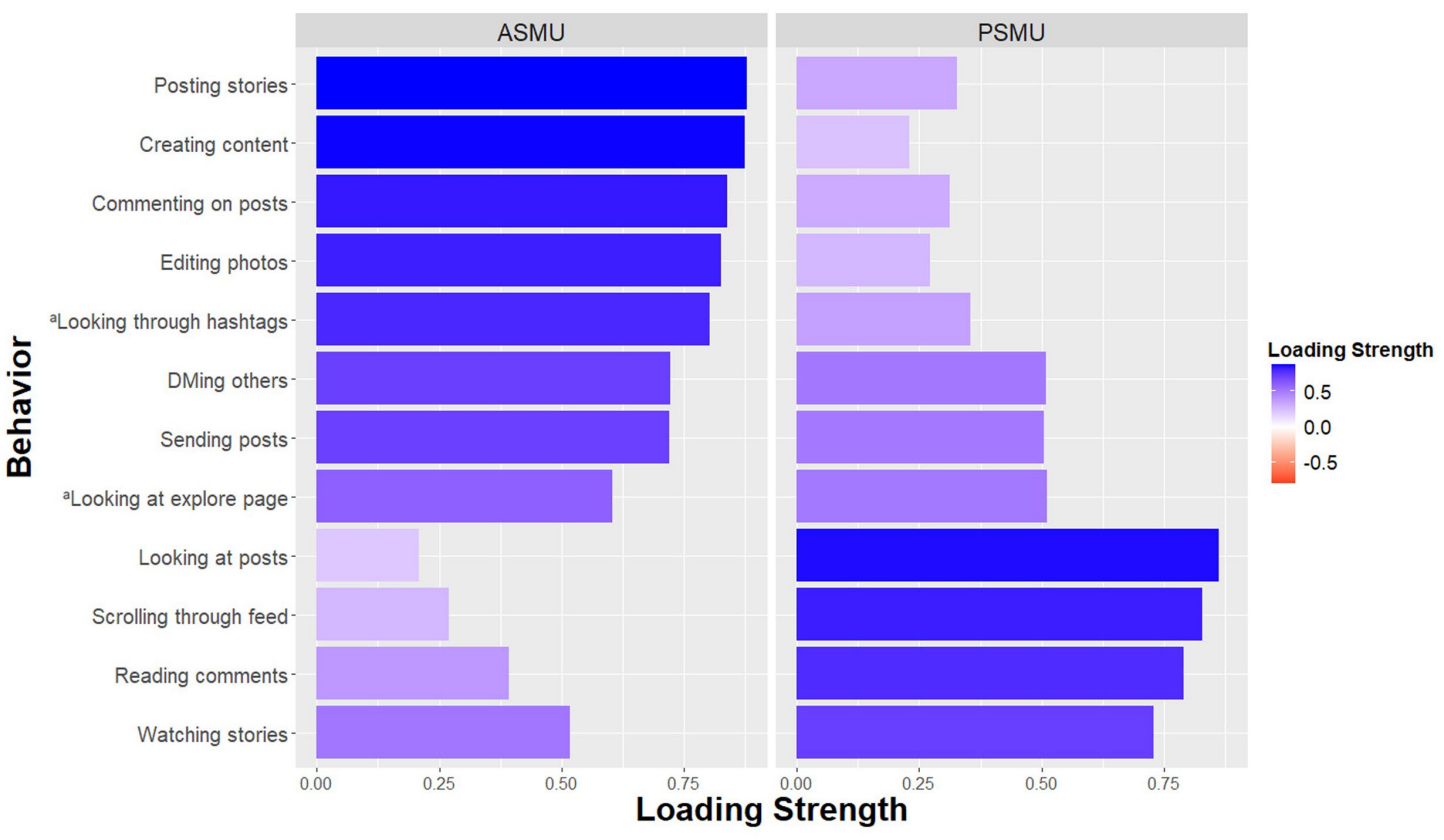
Factor loadings of passive and active items based on a principal components analysis with an orthogonal rotation (*N* = 128). *X*-axis represents the absolute value of factor loading strength while color gradient represents the direction of the loading. ^a^Items excluded from the main study due to concerns with reliability.

We aimed to determine whether the active and passive social media behavior items mapped onto the two-factor structure we expected. A principal component analysis (PCA) with an orthogonal rotation revealed a two-factor solution based on Eigenvalues greater than one which accounted for 78% of the total variance (Figure 1). As expected, all behaviors theorized to be characteristic of ASMU loaded onto one factor. Additionally, four of the six behaviors theorized to be characteristic of PSMU loaded onto the second factor. Two behaviors (“Looking through posts related to a particular hashtag” and “Looking through the explore page”), which we had theorized to characterize passive behavior, loaded more strongly onto the ASMU factor. Thus, we removed these two questions from our final set of questions. With the removal of these two items, the six ASMU questions were reliable at alpha = 0.95 and the four PSMU questions were reliable at alpha = 0.91.

### Discussion

An initial test of the reliability of the modified active and passive social media behaviors from Gerson et al.’s (2017) study revealed a clear differentiation between self-reported ASMU and PSMU. The six items we had hypothesized to characterize ASMU loaded onto one factor, while four out of the six items we had hypothesized would be characteristic of PSMU loaded onto a separate factor. Given that two items we had initially hypothesized to characterize passive behavior shared more variance with other active behaviors, these two items were removed for future analyses.

## Main study

Our main study sought to utilize the ASMU and PSMU items tested in our pre-study in order to directly test whether emotion recognition skill is one process by which social media use relates to mental health. Based upon Ruben et al.’s (2021) findings that ASMU was related to poorer interpersonal skills, and PSMU was related to greater interpersonal skills, we hypothesized the following:

*H1*: Greater self-reported ASMU would be related to poorer emotion recognition skill.

*H2*: Greater self-reported ASMU would be related to poorer mental health (i.e., depressive symptoms, anxiety, and stress).

*H3*: The relationship between self-reported ASMU and mental health would be mediated by emotion recognition skill.

*H4*: Greater self-reported PSMU would be related to greater emotion recognition skill.

*H5*: Greater self-reported PSMU social media use would be related to better mental health (i.e., depressive symptoms, anxiety, and stress).

*H6*: The relationship between self-reported PSMU and mental health would be mediated by emotion recognition skill.

### Method

#### Participants

An online sample of *N* = 150 was recruited *via* the survey platform Prolific and were compensated with $5.00 for their participation in the 30-min study. After removing participants who failed three attention checks and those who did not have at least one of the following social media accounts (Facebook, Instagram, Tik Tok, Snapchat, or Twitter), the final *N* was 139 participants (61.9% female, 36.7% male, 0.14% non-binary or preferred not to say). The sample was 72.5% White, 9.4% Black or African American, 0.7% American Indian or Alaska Native, 10.8% Asian, 0.7% Native American or Pacific Islander, and 5.8% selected “Other” or multiple races. Additionally, 15.1% of participants identified as Hispanic or Latine and the mean age was *M*_age_ = 30.12 (*SD* = 11.27).

#### Procedure

Upon completion of the informed consent, participants were directed to an online survey where they completed a validated measure of emotion recognition ability, the Geneva Emotion Recognition Test - Short Form (GERT-S; Schlegel et al., 2014). Next, participants indicated which of the following social media platforms they used currently: Instagram, Facebook, Twitter, Tik Tok, Snapchat, or none. The survey ended if they selected none. If they indicated that they used at least one of the platforms, they were redirected to the next part of the survey where they were asked to self-report how often they engaged in various active and passive social media behaviors on each social media platform. Finally, they completed the Depression Anxiety Stress Scale (DASS-21, Lovibond and Lovibond, 1995), along with demographic questions.

#### Materials

##### ASMU and PSMU use questions

Participants completed the same ASMU and PSMU questionnaire as in the pre-study, with the exception of the two passive behavior items that were removed due to lack of reliability. Participants were asked to rate on a 1 (*Not often at all*) to 7 (*Very often*) Likert scale how often they engaged in 10 different social media behaviors on five different social media platforms. Six items assessed an individual’s self-reported active behavior on social media (*α* = 0.88) and four items assessed their self-reported passive behavior on social media (*α* = 0.78).

##### Geneva emotion recognition test – short form

The GERT-S (Schlegel et al., 2014) is a dynamic and multimodal performance-based test that measures individual differences in the ability to recognize others’ emotions in the face, body, and voice (α = 0.80; Schlegel and Scherer, 2016). It contains 42 short clips (duration 1–3 s) of 10 actors (five female, all white) seen from the upper portion of their torso and up, uttering syllables with no discernible meaning. After each clip, participants are given the option to select which of 14 emotions the actor was expressing: pride, joy, amusement, pleasure, relief, interest, surprise, anxiety, fear, despair, sadness, disgust, irritation, and anger. For each clip, responses were coded as 0 = incorrect and 1 = correct. Responses were summed, and then divided by the number of total items to give a final score (i.e., proportion correct).

##### Depression anxiety stress scale

The Depression Anxiety Stress Scale (DASS-21) (Lovibond and Lovibond, 1995) is a 21-item self-report measure that assesses depressive symptoms, anxiety, and stress. Participants were asked to rate to what degree each statement applied to them over the past week on a four-point scale (0 = *Did not apply to me at all* and 3 = *Applied to me very much, or most of the time*) with a higher score indicating more severe symptoms. According to the DASS Scoring Manual, we grouped scores on depression, anxiety, and stress into two categories of “Severe” and “Non-severe.”^3^ The DASS-21 has shown strong psychometric properties as well as the ability to discriminate between the constructs of depression, anxiety, and stress (Clara et al., 2001).

### Results

We first examined whether the psychometric properties of our social media behavior items replicated with a second sample of participants. Although the mean endorsement of each behavior item was slightly different than our pre-study, participants reported the exact same ordering of mean scores of individual active and passive behaviors with the exception of two items (i.e., participants indicated that, on average, they “Scroll through my feed” to a slightly greater degree than they “Look at friends’ or strangers’ posts/photos”; Table 1).

Once again, we performed a factor analysis on the various active and passive social media behaviors using a PCA with an orthogonal rotation. A two-factor solution was retained which accounted for 63% of the total variance. Replicating our first sample, all behaviors theorized to be characteristic of ASMU loaded onto one factor and all behaviors theorized to be characteristic of PSMU loaded onto another. Thus, we proceeded to form a composite score for each participant of their total mean ASMU (alpha = 0.88), and total PSMU (alpha = 0.78) across all five social media platforms for subsequent analyses.

In order to examine the relationships between social media use, emotion recognition, and mental health, we first examined the zero-order correlations between these variables (Table 2).^4^ Participants who reported greater ASMU were also more likely to report greater PSMU (*r* = 0.56, *p* < 0.001). Participants’ ASMU score correlated positively with anxiety (*r* = 0.24, *p* = 0.005) and stress (*r* = 0.20, *p* = 0.017), as well as negatively with emotion recognition skill (*r* = −0.28, *p* < 0.001). On the other hand, there was a small relationship between participants’ PSMU and anxiety (*r* = 0.14, *p* = 0.099) and PSMU was negatively related to emotion recognition skill (*r* = −0.17, *p* = 0.044). All of these relationships reported above were small to moderate in magnitude. Finally, neither depression, anxiety, nor stress appeared to be significantly related to emotion recognition skill (*r*’s < −0.11, *p*’s > 0.229).

**Table 2:** Descriptive statistics and zero-order correlations for ASMU and PSMU composites, mental health, emotion recognition skill, and sex (*N* = 139).

Variables	*M* (*SD*)	2	3	4	5	6	7
1. ASMU composite	2.77 (1.23)	0.56^***^	−0.01	0.24^**^	0.21^*^	−0.28^***^	−0.09
2. PSMU composite	4.33 (1.20)		−0.02	0.14^†^	0.09	−0.17^*^	0.06
3. Depression	Severe *N* = 39, Non-severe *N* = 94			0.40^***^	0.28^**^	0.07	0.13
4. Anxiety	Severe *N* = 28, Non-severe *N* = 105				0.55^***^	−0.11	0.10
5. Stress	Severe = 18, Non-severe *N* = 115					0.00	0.15
6. Emotion recognition skill	0.61 (0.12)						0.08
7. Sex	Male *N* = 51, Female *N* = 86						

Male was coded as 0, female was coded as 1. Emotion recognition skill scores could range from 0 to 1. ^†^*p* < 0.10, ^*^p < 0.05, ^**^p < 0.01, ^***^p < 0.001.

#### Mediation models

**Figure 2:**
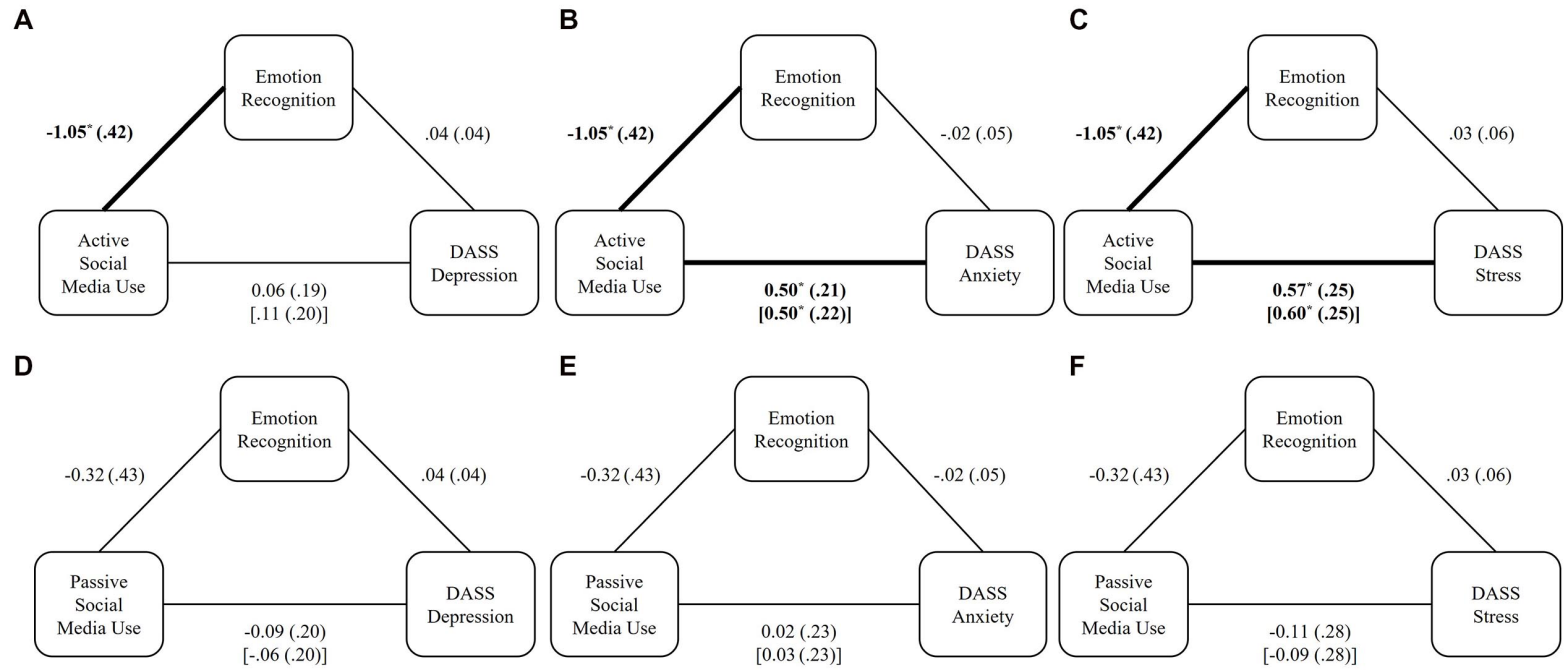
Mediation models of the hypothesized relationship between ASMU and PSMU, emotion recognition, and mental health outcomes. **(A)** Relationship between ASMU and depression as mediated by emotion recognition. **(B)** Relationship between ASMU and stress as mediated by emotion recognition. **(C)** Relationship between ASMU and anxiety as mediated by emotion recognition. **(D)** Relationship between PSMU and depression as mediated by emotion recognition. **(E)** Relationship between PSMU and stress as mediated by emotion recognition. **(F)** Relationship between PSMU and anxiety as mediated by emotion recognition. Unstandardized coefficients are presented with standard errors in parentheses. Direct effects are reported in brackets. For models **(A–C)**, PSMU and sex were entered as covariates. For models **(D–F)**, ASMU and sex were entered as covariates. Paths b, c, and c’ in each mediation model are expressed in a log-odds metric. Paths significant at *p* < 0.05 are bolded and marked with *.

We next tested whether a mediating relationship was supported between our variables of interest. Specifically, we hypothesized that more self-reported ASMU would be related to poorer emotion recognition skill and a greater likelihood for experiencing severe depression, anxiety, and stress. Additionally, we hypothesized that more self-reported PSMU would be associated with greater emotion recognition skill and a lesser likelihood of experiencing severe depression, anxiety, and stress. We conducted a series of six meditations (see Figure 2) to test these hypotheses using Hayes PROCESS macro in SPSS (Hayes and Rockwood, 2017). Because ASMU and PSMU were strongly correlated with each other, we controlled for the opposing social media variable, along with sex, in each mediation model.

Models A, B, and C (Figure 2) show the relationship between self-reported ASMU and depression, anxiety, and stress through emotion recognition skill, controlling for PSMU and participant sex. These models revealed support for H1; individuals who reported greater ASMU displayed poorer emotion recognition skill (*B* = −1.05, *p* = 0.014). In support of H2, for every one unit increase in ASMU, the odds of experiencing severe anxiety increased by 1.64 (*p* = 0.021) and the odds of experiencing severe stress increased by 1.82 (*p* = 0.017). However, greater ASMU was not significantly related to a greater likelihood of experiencing severe depression (*p* = 0.574). We also did not find evidence to suggest that individuals’ emotion recognition skills were significantly related to any mental health variables (*p*’s < 0.640). Following, we did not find evidence to support H3, that emotion recognition skill mediated the relationship between ASMU and depression (indirect effect *B* = −0.04, *SE* = 0.05), anxiety (indirect effect *B* = 0.02, *SE* = 0.07), or stress (indirect effect *B* = −0.03, *SE* = 0.09).

Models D, E, and F (Figure 2), show the relationship between self-reported PSMU and depression, anxiety, and stress through emotion recognition skill, controlling for ASMU and participant sex. Contrary to what was hypothesized, PSMU was not related to emotion recognition skill (H4; *B* = −0.32, *p* = 0.455), nor any of our mental health variables (*p’s >* 0.740) as was predicted by H5. Consequently, we did not find evidence to support H6, that emotion recognition skill mediated the relationship between PSMU and any mental health outcome.

### Discussion

The New York Times recently documented a small group of teenagers in New York City who are trading in their smartphones for flip phones because of concerns over the relationship between social media and mental health (Vadukul, 2022). Counterculture revolutions like this are not uncommon in 2023. The reliance on smartphones and addiction to social media has certainly drawn much attention and concern (Dixon, 2023) and psychologists are still working to understand how the utilization of these platforms impacts psychosocial outcomes such as interpersonal skills and individual’s overall mental health. The present study sought to aid some of these central questions by testing whether ASMU and PSMU relate to emotion recognition and mental health. Our modified self-report measure of social media use was able to reliably distinguish between social media behaviors that are more “active” and those that are more “passive.” Further, these two social media use styles displayed unique relationships with emotion recognition skills and mental health.

As predicted, our results confirmed that for every one unit increase in ASMU, the likelihood of experiencing severe anxiety increased by 64% and the likelihood of experiencing severe stress increased by 82%. However, ASMU was not significantly related to a greater likelihood of experiencing severe depression. Due to the inherent risks that come with creating and engaging with social media content (e.g., actual or perceived negative peer judgment), these findings could indicate that taking the more active role of creating and engaging with content may be a source of anxiety and stress. Alternatively, it is possible that individuals who are severely anxious or stressed may engage in social media more actively as a form of escapism, or to find opportunities to co-ruminate or find acceptance with others (O’Day and Heimberg, 2021; Jones and Heerey, 2022). Future research should seek to explore these findings further using experimental research designs to determine the causal relationships between ASMU, emotion recognition, and mental health.

Contrary to what was expected, our results do not suggest that engaging in PSMU, such as reading through the comments on other peoples’ posts, is related to less severe depression, anxiety, or stress. This is encouraging information, as previous studies often show how objective time spent on social media is related to poorer mental health (Lin et al., 2016; Keles et al., 2020). Our null relationships between PSMU and various mental health symptoms highlights that how one spends their time matters. That is, when a user is contributing to content on social media platforms (e.g., posting, commenting, sharing), these might be important risk factors to consider when evaluating mental health symptoms such as severe anxiety and stress, while viewing others’ posts and content appears to be unrelated to mental health.

Regarding emotion recognition skill, we found that the more individuals self-reported engaging in ASMU (e.g., “Commenting on friends’/strangers’ posts”), the poorer were their emotion recognition skills on an objective test of emotion recognition. In line with the *reduction hypothesis* (Kraut et al., 1998; Nie, 2001), these findings may reflect that actively engaging with social media comes at the expense of forming and growing face-to-face social relationships – a process that may be central to the development of emotion recognition skills as it allows one to practice and receive feedback on the correctness of their perception of others’ emotions. However, given that our data was correlational, it is also equally plausible that those who initially experience difficulty in recognizing the emotional expressions of others may be more inclined to be more active on social media, as face-to-face interactions could feel more strenuous or less rewarding than online relationships. It is also possible that a third variable, such as how self-focused or narcissistic an individual is, may impact both emotion recognition ability and ASMU. Future research should rule out these third variables and examine the causal paths by which social media use impacts emotion recognition ability.

In contrast to what we expected, and Ruben et al. (2021) findings, we did not find evidence to support PSMU (e.g., “Looking at friends’ or strangers’ posts/photos) was related to greater emotion recognition ability. One possible explanation for why these two variables were unrelated in the present study can be understood within the context of the Cues Filtered Out Theory (Culnan and Markus, 1987; Keiser, 1987), which argues that certain social media platforms limit the amount of nonverbal cues available to users (e.g., vocal tone, posture, gesture, etc.). Even though passive social media users are able to practice their emotion recognition skills online, they may only be developing this skill for more exaggerated *static* emotional expressions (e.g., photographs on Instagram) or *posed* emotional expressions (e.g., videos on TikTok), opposed the natural and dynamic emotional expressions that occur in face-to-face interactions. Thus, while Ruben et al. found that PSMU was associated with greater emotion recognition skill on a measure of static emotion recognition (i.e., photographs from the DANVA-2AF), the present study did not extend these findings to a measure of dynamic emotion recognition skill (i.e., videos in the GERT-S).

Finally, we did not find evidence to suggest that emotion recognition ability is one process that explains the relationship between ASMU/PSMU and mental health. While the present investigation focused on emotion recognition as a central interpersonal skill, it is possible that there may be different interpersonal skills, such as social communication (i.e., encoding) abilities, that may be more likely to mediate the relationship between social media use styles and mental health. Additionally, there are other possible mediating mechanisms that could help explain how ASMU is related to a greater likelihood of experiencing severe anxiety and stress, such as personality traits and self-esteem. Future research should continue to explore these possible pathways in order to establish possible avenues for mental health interventions.

### Limitations and future directions

When considering the generalizability of these findings, it is important to note that both studies utilized data generated from paid online samples. While online samples have been shown to be representative of the general population across certain demographic characteristics such as race and ethnicity (Buhrmester et al., 2011) and most psychological constructs including depression and anxiety (Shapiro et al., 2013; Arditte et al., 2016), there are a few notable exceptions that apply to the current work. Specifically, online samples seem to represent the stereotypical frequent Internet user (Goodman and Paolacci, 2017; McCredie and Morey, 2019) as they tend to be younger, more educated, less religious and more liberal than the general population. Thus, our sample likely captures the relationships between social media use and various outcomes among people who are more familiar and savvy with social media compared to the general population.

Although this study was the first to examine the relationships between social media use styles, emotion recognition, and mental health outcomes together in one model, these data are correlational in nature. As mentioned previously, with cross-sectional data we are unable to determine the directionality or causality of the variables in question. Future research would benefit from collecting longitudinal data as well as experimental data to determine the directionality and causality of the relationships found (see Ruben et al., 2021 for a discussion of potential experimental designs for examining the impact of “active” and “passive” social media use on outcomes).

Another limitation of this study is the way in which mental health was measured. While the DASS provides important information about general depression, anxiety, and stress, this measure is not diagnostic and does not provide a comprehensive evaluation of participants’ mental health. Further, because our sample of participants was not equally distributed into severe and non-severe for each DASS subscale, our mediation models faced a reduction in power. The use of diagnostic measures in the future, as well as sampling a greater number of participants with severe anxiety, depression, and stress, would provide clearer information about psychopathology of internalizing disorders as well as other disorders that are often associated with social media, such as eating disorders and body dysmorphia (Santarossa and Woodruff, 2017).

Finally, while a strength of the current paper was modifying the only measure of ASMU and PSMU that we are aware of to reliably generalize across multiple social media platforms, the aim of the present study was *not* to validate this measure as an entirely new scale. Future research should continue exploring the psychometric properties of this measure and refining it to fit their own specific research questions regarding social media use. Measures such as this are short, easily distributable, and greatly enhance the rigor of social media studies that better allow researchers to gain new knowledge of and develop important psychological interventions surrounding social media use that improve well-being and social interactions.

In line with this recommendation, we also wish to draw the reader’s attention to a few recent conceptual frameworks that address the limitations of the active and passive social media distinction (Meier and Krause, 2022; Verduyn et al., 2022). While this framework has many strengths, such as moving social media research past simple screentime usage, being robust and generalizable across existing and future social media platforms and providing heuristic value for translational research practices (Meier and Krause, 2022), various critiques have also been raised. For instance, Verduyn et al. (2022) argue that it is not the active or passive behaviors that may impact individuals mental health per say, but the reciprocity and communion (i.e., warmth) of the exchanges individuals engage in on social media. These more targeted and thoughtful approaches to measuring how individuals use social media will certainly help us better understand the impact of social media on individuals health and well-being.

## Conclusion

While the field of social media research still largely relies on measures of global time spent on social media to answer relevant research questions, the present research illuminates the problems with conclusions drawn from this approach. It may not always be spending time on social media that is associated with negative interpersonal outcomes, but *how* one spends their time online that matters. The current research demonstrates the complexity with measuring social media use by modifying an existing multi-item measure of ASMU and PSMU to capture different social media behavioral styles that is generalizable across multiple social media platforms. This work clearly demonstrated that ASMU and PSMU are unique styles of social media engagement that relate to psychosocial outcomes such as emotion recognition and mental health in disparate ways. Specifically, more ASMU social media use was related to more severe anxiety and stress as well as poorer emotion recognition skill, while PSMU was unrelated to these important psychological constructs.

We find these results both important, and encouraging, as they suggest that blanket statements regarding the negative impacts of social media use may not hold for those who use social media in a passive, observational manner. However, it is important to address the concerning finding that more active social media users are at greater risk for severe anxiety and stress. Although our results do not necessarily suggest that active social media use is a *cause* of anxiety and stress, the link between the two certainly merits consideration by platform providers and regulatory bodies to ensure their users are appropriately supported while utilizing their platforms. For instance, if one of the mechanisms by which ASMU is related to stress and anxiety is through negative feedback/judgment to one’s content, then platform providers could seek to better police negative dialogue between users. Platform providers may even consider incentivizing more positive engagement and dialogue between users to combat the stress and anxiety generated from a *lack* of positive engagement with one’s content (e.g., not receiving many likes or comments on one’s post). WorkHuman (2019), a platform explicitly designed to incentivize employees to give gratitude and recognition to one another in the workplace, serves as an example of this kind of intervention. As the field of social media research continues to move forward, adopting measures of social media use that capture what individuals are doing online, opposed to how much time they spend online, will only further our ability to develop interventions and social media regulations that enhance individuals’ psychological well-being in a world increasingly filled with social media engagement.

## Data availability statement

The datasets presented in this study can be found in online repositories. The names of the repository/repositories and accession number(s) can be found at: https://osf.io/zkupq.

## Ethics statement

The studies involving human participants were reviewed and approved by University of Maine IRB. The patients/participants provided their written informed consent to participate in this study.

## Author contributions

ES and MS contributed to conception and design of the study. MS organized the database and performed the statistical analysis. ES wrote the first draft of the manuscript. MS, AW, and MR wrote sections of the manuscript. MR led supervision of the research. All authors contributed to manuscript revision, read, and approved the submitted version.

## Conflict of interest

The authors declare that the research was conducted in the absence of any commercial or financial relationships that could be construed as a potential conflict of interest.

## Publisher’s note

All claims expressed in this article are solely those of the authors and do not necessarily represent those of their affiliated organizations, or those of the publisher, the editors and the reviewers. Any product that may be evaluated in this article, or claim that may be made by its manufacturer, is not guaranteed or endorsed by the publisher.

## Footnotes

^1^The researchers did not report whether the remaining associations supported a null-effect, or supported the alternative hypothesis that ASMU was related to greater ill-being and PSMU was related to less ill-being.

^2^The categories of active and passive social media user were not mutually exclusive, such that a person could be both a high active user and a high passive user of social media.

^3^As requested by reviewers, we also ran all mediational analyses below treating each DASS variable as continuous. The only change in significance from this approach was the direct effect of ASMU on stress, which became non-significant (*B* = 1.33. *p* = 0.145).

^4^See Supplementary Table S1, for the relationships between the specific active and passive behavioral items and each DASS variable and emotion recognition skill.
