# Supplementary material for: Should I post? The relationships among social media use, emotion recognition, and mental health
Source: Front Psychol. 2023 May 23;14:1161300. doi: 10.3389/fpsyg.2023.1161300 (PMC10242173; doi:10.3389/fpsyg.2023.1161300)
Supplement: Supplementary file 1 [file Table_1.docx]

**Supplemental Material**

Table 1. The Independent Contribution of Social Media Behaviors on Mental Health and Emotion Recognition Skills (*N* = 139).

|  | Social Media Behaviors | Depression  *r* (*r*_partial_) | Anxiety  *r* (*r*_partial_) | Stress  *r* (*r*_partial_) | Emotion Recognition  *r* (*r*_partial_) |
| --- | --- | --- | --- | --- | --- |
| Active | Editing photos | -.03 (-.01)^1^ | .11 (.02)^1^ | .10 (.06)^1^ | -.25^**^ (-.22^*^)^1^ |
|  | Commenting on posts | -.03 (.00)^1^ | .16^†^ (.14)^1^ | .17^*^ (.22^*^)^1^ | -.29^**^ (-.25^**^)^1^ |
|  | Posting Stories | -.05 (-.04)^1^ | .21^*^ (.14)^1^ | .10 (.09)^1^ | -.25^**^ (-.22^*^)^1^ |
|  | Creating Content | -.03 (-.02)^1^ | .14 (.14)^1^ | .09 (.11)^1^ | -.28^**^ (-.25^**^)^1^ |
|  | Sending posts | -.07 (-.11)^1^ | .24^**^ (.09)^1^ | .17^†^ (.07)^1^ | -.20^*^ (-.16^†^)^1^ |
|  | DMing others | .07 (.08)^1^ | .13 (.07)^1^ | .14 (.12)^1^ | -.11 (-.03)^1^ |
| Passive | Looking at posts | .04 (.06)^2^ | .08 (-.02)^2^ | .01 (-.12)^2^ | -.03 (.07)^2^ |
|  | Reading comments | -.09 (-.08)^2^ | .09 (-.02)^2^ | .10 (-.03)^2^ | -.17^*^ (-.10)^2^ |
|  | Watching stories | -.03 (-.03)^2^ | .14 (.04)^2^ | .06 (-.06)^2^ | -.25^**^ (-.17^†^)^2^ |
|  | Scrolling through feed | -.01 (.00)^2^ | .13 (-.01)^2^ | .08 (-.04)^2^ | -.05 (.08)^2^ |

*Note.* ^1^ Controlling for passive behavior composite. ^2^ Controlling for active behavior composite. ^†^*p* < .10, ^*^ *p* < .05, ^**^ *p* < .01, ^***^ *p* < .001
